# Endophytic Actinobacteria and the Interaction of *Micromonospora* and Nitrogen Fixing Plants

**DOI:** 10.3389/fmicb.2015.01341

**Published:** 2015-12-01

**Authors:** Martha E. Trujillo, Raúl Riesco, Patricia Benito, Lorena Carro

**Affiliations:** Departamento de Microbiología y Genética, Universidad de SalamancaSalamanca, Spain

**Keywords:** *Micromonospora*, legumes, PGPB, actinorhizal, endophytic, nodule

## Abstract

For a long time, it was believed that a healthy plant did not harbor any microorganisms within its tissues, as these were often considered detrimental for the plant. In the last three decades, the numbers of studies on plant microbe-interactions has led to a change in our view and we now know that many of these invisible partners are essential for the overall welfare of the plant. The application of Next Generation Sequencing techniques is a powerful tool that has permitted the detection and identification of microbial communities in healthy plants. Among the new plant microbe interactions recently reported several actinobacteria such as *Micromonospora* are included. *Micromonospora* is a Gram-positive bacterium with a wide geographical distribution; it can be found in the soil, mangrove sediments, and freshwater and marine ecosistems. In the last years our group has focused on the isolation of *Micromonospora* strains from nitrogen fixing nodules of both leguminous and actinorhizal plants and reported for the first time its wide distribution in nitrogen fixing nodules of both types of plants. These studies have shown how this microoganism had been largely overlooked in this niche due to its slow growth. Surprisingly, the genetic diversity of *Micromonospora* strains isolated from nodules is very high and several new species have been described. The current data indicate that *Micromonospora saelicesensis* is the most frequently isolated species from the nodular tissues of both leguminous and actinorhizal plants. Further studies have also been carried out to confirm the presence of *Micromonospora* inside the nodule tissues, mainly by specific *in situ* hybridization. The information derived from the genome of the model strain, *Micromonospora lupini*, Lupac 08, has provided useful information as to how this bacterium may relate with its host plant. Several strategies potentially necessary for *Micromonospora* to thrive in the soil, a highly competitive, and rough environment, and as an endophytic bacterium with the capacity to colonize the internal plant tissues which are protected from the invasion of other soil microbes were identified. The genome data also revealed the potential of *M. lupini* Lupac 08 as a plant growth promoting bacterium. Several loci involved in plant growth promotion features such as the production of siderophores, phytohormones, and the degradation of chitin (biocontrol) were also located on the genome and the functionality of these genes was confirmed in the laboratory. In addition, when several host plants species were inoculated with *Micromonospora* strains, the plant growth enhancing effect was evident under greenhouse conditions. Unexpectedly, a high number of plant-cell wall degrading enzymes were also detected, a trait usually found only in pathogenic bacteria. Thus, *Micromonospora* can be added to the list of new plant-microbe interactions. The current data indicate that this microorganism may have an important application in agriculture and other biotechnological processes. The available information is promising but limited, much research is still needed to determine which is the ecological function of *Micromonospora* in interaction with nitrogen fixing plants.

## Introduction

Bacteria, archaea, and viruses are present in every niche present in our planet and have a great impact on other forms of life. Since the appearance of plants on Earth, their capacity to adapt to different ecosystems and their evolutionary process have inherently been associated to microorganisms (Reid and Greene, 2012).

Microbial communities present in soil account for the richest reservoir of biological diversity in our planet (Berendsen et al., 2012). Microorganisms that live in the rhizosphere, the soil region influenced by plant roots, are of great importance as this is where most plant-microbe interactions occur (Schenk et al., 2012) and this complex plant-associated microbial community is for the most part beneficial to the plant (Berendsen et al., 2012). Despite the importance of microorganisms for plants, these extremely complex microbial communities have remained largely uncharacterized mainly due to our lack of culturing most microorganisms under laboratory conditions (Schenk et al., 2012). Fortunately, our awareness of mutually beneficial relationships and their potential application in biotechnological processes is expanding, in part due to the new sequencing technologies and information derived from their use.

Microbes that interact with plants are termed rhizospheric or endophytic depending on their localization outside or inside the plant, respectively, and many endophytes originate from the rhizosphere or phyllosphere (Dudeja et al., 2012). These organisms can accelerate seed germination, promote plant establishment under adverse conditions, enhance plant growth or prevent pathogen infections (Hurek et al., 2002; Ryan et al., 2008). Thus, a complex and invisible ecosystem sustains plant growth and health (Reid and Greene, 2012). The potential application of beneficial microbes in different fields (e.g., agriculture, biotechnology, medicine, etc.) is immense provided progress is made in understanding these complex plant-microbe interactions in a global context.

Hitherto, plant associated Gram-negative bacteria are the best studied given their relative facility to be recovered from internal plant tissues and also because mutants can be easily generated for interaction studies (Francis et al., 2010). However, many Gram-positive bacteria included in the phyla *Firmicutes* and *Actinobacteria* (e.g., *Bacillus, Micromonospora, Streptomyces*, etc.) have excellent biocontrol, plant growth-promoting and bioremediation activities. In addition, several characteristics observed including pigment and spore production, biosynthesis of secondary metabolites and unique lifestyles present in these microorganisms can be advantageous for different biotechnological applications, including agriculture.

In this review, the diversity and interaction between actinobacteria and plants will be discussed, focusing on their ecological aspects and potential applications in agriculture. The second part of this revision will focus on the specific interaction of the genus *Micromonospora* with nitrogen fixing plants.

## Plant-associated actinobacteria

Actinobacteria represent approximately 20–30% of the rhizospheric microbial community (Bouizgarne and Ben Aouamar, 2014). They are Gram-positive and show a wide morphological spectrum ranging from unicellular organisms to branching filaments that form a mycelium. A unique feature is their high guanine plus cytosine content (>50%) in their genome. These microorganisms are for the most part saprophytic, soil-dwelling organisms with an important role in the turnover of organic matter. In addition, many species are sporulated and spend the majority of their life cycles as semidormant spores (Coombs and Franco, 2003a).

Several taxa are well-known to interact with plants and these include examples of both endophytic and plant-pathogenic species. The first actinobacterial endophyte isolated, *Frankia* (Callaham et al., 1978), is a nitrogen-fixing microorganism that induces nodulation on several angiosperm plant families and has received a lot of attention due to its role in the nitrogen economy of its hosts (Verma et al., 2009). Several plant-pathogenic taxa include *Streptomyces acidiscabies, Streptomyces europaeiscabiei, Streptomyces scabies*, and *Streptomyces turgidiscabies* which cause potato scab (Loria et al., 2006; Bignell et al., 2010)*; Clavibacter michiganensis* with several subspecies and pathogen for alfalfa (*C. michiganensis* subsp. *insidiosus*), maize (*C. michiganensis* subsp. *nebraskrensis*), potato (*C. michiganensis* subsp. *michiganensis*) and wheat (*C. michiganensis* subsp. *tessellarius*); *Leifsonia xyli* subsp. *xyli* which causes ratoon stunting disease of sugarcane (Young et al., 2006); *Curtobacterium flaccumfaciens* which affects several *Phaseolus* and *Vigna* species, *Beta vulgaris* species (red and sugar beet), *Ilex opaca* (American holly), *Tulipa* species (tulips), and *Euphorbia pulcherrima* (poinsettia) (Saddler and Messenber-Guimaraes, 2012); *Rathayibacter iranicus* and *Rathayibacter tritici* which cause gumming in several grasses and wheat (Evtushenko and Dorofeeva, 2012).

In the last decade, many reports on the isolation and diversity of plant-associated and endophytic actinobacteria from wild plants and crops have been published. In many of these studies, a neutral or a plant growth promotion effect was observed. The isolation and identification of actinobacteria in healthy internal root tissues of wheat was reported by Coombs and Franco (2003a); these authors further demonstrated the colonization of germinating wheat by one of the isolated strains, *Streptomyces* sp. EN27 (Coombs and Franco, 2003b). A *Streptomyces* strain, WYEC108, isolated from linseed rhizosphere soil in Great Britain (Crawford et al., 1993) was able to colonize the roots of *Pisum sativum*, increased the number and size of root nodules, and enhanced the assimilation of iron and other nutrients by the plant (Tokala et al., 2002). Several actinobacterial strains recovered from wild plants adapted to poor soil and severe climate conditions of the Algerian Sahara desert were reported by Goudjal et al. (2013). Some of these strains produced the auxin indol acetic acid (IAA), which promoted seed germination and root elongation when tomato seeds were treated with bacterial supernatants.

The search of endophytic actinobacteria as biological control agents of plant disease is also of interest given their ability to colonize healthy plant tissues and produce antibiotics *in situ* (Kunoh, 2002; Cao et al., 2004). Maize (*Zea mays*), an important crop cultivated in many countries, especially in tropical areas, was also screened for the presence of bioactive actinobacteria (de Araújo et al., 2000). Endophytic streptomycetes isolated from healthy banana plants (*Musa* sp.), were studied for the ability to produce antifungal molecules that inhibited the growth of *Fusarium oxysporum*, which causes fusarium wilt (Cao et al., 2005). Similarly, *Streptomyces* strains were isolated from tomato and native plants of the Algerian Sahara and screened for biocontrol activity against *Rhizotocnia solani* (Goudjal et al., 2014).

Several studies have focused on the diversity and distribution of actinobacterial communities in plants, these works have provided information about the most common taxa found, e.g., the genus *Streptomyces*, but have also discovered new plant-actinobacteria associations as those represented by the interaction *Micromonospora*-nitrogen fixing plants.

Members of the genera *Microbispora, Micromonospora, Nocardia, Streptosporangium*, and *Streptoverticillium* were recovered from the surface of sterilized roots of different plant species in Italy (Sardi et al., 1992) and of maize in Brazil (de Araújo et al., 2000). Interestingly, the genus *Microbispora* was the most abundant genus recovered in maize (44%), followed by *Streptomyces* and *Streptosporangium*. A diverse collection of 11 native Korean plants were screened for the presence of endophytic actinobacteria. *Streptomyces* was the most common taxon accounting for almost 50% of the strains isolated and followed by the genera *Microbacterium, Microbispora, Micrococcus, Micromonospora, Rhodococcus*, and *Streptacidiphilus*. Single isolates representing the genera *Arthrobacter, Dietzia, Herbiconiux, Kitasatospora, Mycobacterium, Nocardia, Rathayibacter*, and *Tsukamurella* were also recovered (Kim et al., 2012).

Kaewkla and Franco (2013) demonstrated the high diversity of actinobacterial strains distributed in native Australian plants using highly designed isolation protocols which included low concentration isolation media, plating larger quantities of plant sample and long incubation times (up to 16 weeks). These authors reported the isolation of >500 actinobacterial strains that were identified in 16 different genera. Again, the genus *Streptomyces* accounted for >60% of the isolates.

Although the percentage of plant species sampled at present is very low, medicinal plants have received special attention given their importance as potential reservoirs of actinobacterial communities that produce compounds with biotechnological application. Qin et al. (2009, 2012) conducted a thorough study screening medicinal plants growing in the tropical rain forests in Xishuangbanna, China. These authors focused on the isolation of non-streptomycetes and found that the genus *Pseudonocardia* was the predominant taxon, followed by *Nocardiopsis, Micromonospora*, and *Streptosporangium* while almost 25% of the strains could not be identified at the genus level. An in depth analysis of the plant *Maytenus austroyunnanensis* applying culture- dependent and independent methods revealed an immense diversity reporting genera such as *Actinostreptospora, Amnibacterium, Catenuloplanes, Quadrisphaera*, and *Pseudokineococcus* which were previously unknown to reside inside plant tissues (Qin et al., 2012).

A list of endophytic and plant-associated actinobacteria recovered from different plant species and their potential application in agriculture is presented in Table 1.

**Table 1:** **Endophytic and plant-associated actinobacteria reported in the literature**.

**Genus**	**Host plant**	**Isolation source**	**References**	**Potential use**
Frankia^*^	*Comptonia*	Root nodule	Callaham et al., 1978	Nitrogen fixation
*Actinosynnema*	*Grass blade*	–	Hasegawa et al., 1978	Not determined
*Streptomyces*	*Allium porrum, Amaryllis belladona, Betula pendula, Brassica oleracea, Calluna vulgaris, Chelidonium majusCichonum intybus, Euphorbia* sp., *Fragaria vesca, Lactuca scariola, Quercus* sp.*, Rubus idaeus*	Roots	Sardi et al., 1992	Not determined
*Streptomyces*	*Linum usitatissimum*	Rhizosphere soil	Crawford et al., 1993	Growth promotion
*Microbispora, Streptomyces, Streptosporangium*	*Zea mays*	Roots	de Araújo et al., 2000	Biocontrol
*Microbispora, Micromonospora, Nocardioides, Streptomyces*	*Triticum aestivum*	Roots and leaves	Coombs and Franco, 2003a	Biocontrol agent
*Streptomyces*	*Licopersicon esculentum*	Roots	Cao et al., 2004	Biocontrol
*Streptomyces, Streptoverticillium, Streptosporangium*	*Musa* sp.	Roots	Cao et al., 2005	Biocontrol of *Fusarium oxysporum*
*Agromyces, Microbacterium*	*Retama taetam, Ononis natrix, Argyrolobium uniflorum, Astragalus armatus*	Root nodules	Zakhia et al., 2006	Not determined
*Actinoplanes, Micromonospora, Streptomyces*	*Cucumis sativus*	Roots	El-Tarabily et al., 2009	Biocontrol; growth promotion
*Microbispora, Nocardia Sacchromonospora, Streptomyces, Streptosporangium, Streptoverticillium*	*Azadirachta indica*	Leaves, stems, roots	Verma et al., 2009, 2011	Siderophore production; biocontrol
*Pseudonocardia, Nocardiopsis, Micromonospora, Streptosporangium*	*Phyllanthus urinaria, Kadsura heteroclita, Maesa indica, Rauvolfia verticillata, Paris yunnanensis, Maytenus austroyunnanensis, Gloriosa superba, Scoparia dulcis, Tadehagi triquetrum, Goniothalamus* sp., *Cephalotaxus* sp., *and Azadirachta* sp.	Leaves, stems, roots	Qin et al., 2009	Secondary metabolites
*Arthrobacter, Dietzia Herbiconiux, Intrasporangium, Kitasatospora, Microbacterium, Microbispora, Micrococcus Micromonospora Mycobacterium, Nocardia Rathayibacter, Rhodococcus, Streptacidiphilus, Streptomyces, Tsukamurella*	*Artemisia princeps, Capsella bursa-pastoris, Chelidonium majus, Conyza canadensis, Erigeron annuus, Iris rossii, Lamium purpureum, Physostegia virginiana, Rudbeckia bicolor, Setaria viridis, Viola mandshurica*	Roots	Kim et al., 2012	Growth promotion, biocontrol
*Actinomadura, Amycolatopsis, Cellulosimicrobium, Gordonia, Glycomyces, Janibacter, Jiangella, Microbacterium, Micromonospora, Mycobacterium, Nocardia, Nocardiopsis, Nonomuraea, Plantactinospora, Polymorphospora, Promicromonospora, Pseudonocardia, Streptosporangium, Streptomyces, Saccharopolyspora, Tsukamurella*	*Maytenus austroyunnanensis*	Root, stem, leaves	Qin et al., 2012	Not determined
*Actinomadura, Actinomycetospora, Actinopolymorpha, Amycolatopsis, Gordonia, Kribbella, Micromonospora, Nocardia, Nocardioides, Nocardiopsis, Nonomuraea, Polymorphospora, Promicromonospora, Pseudonocardia, Streptomyces, Williamsia*	*Callitris preissii, Eucalyptus camaldulensis, Eucalyptus microcarpa, Pittosporum phylliraeoides*	Leaves, stems, roots	Kaewkla and Franco, 2013	Not determined
*Actinomadura, Kibdelosporangium, Kitasatospora, Micromonospora, Microtetraspora, Nocardia, Nocardioides, Nocardiopsis, Promicromonospora, Pseudonocardia, Saccharopolyspora, Streptoalloteichus, Streptomyces*	*Achillea fragrantissima, Artemisia judaica, Centaurea scoparia, Chiliadenus montanus, Echinops spinosus, Iphiona mucronata, Pulicaria crispa, Scariola orientalis, Seriphidium herba-album, Tanacetum sinaicum*	Not specified	El-Shatoury et al., 2013	Growth promotion
*Streptomyces*	*Cleome arabica, Solanum nigrum, Astragallus armatus, Aristida pungens, Panicum turgidum*	Roots	Goudjal et al., 2013, 2014	Biocontrol, IAA production, growth promotion
*Amycolatopsis, Isoptericola, Micromonospora, Microbispora, Nocardia, Nonomuraea, Promicromonospora, Pseudonocardia, Streptomyces*	*Acacia auriculiformis, Bauhinia purpurea, Canavalia gladiate, Cassia fistula, Clitoria ternatea, Erythrina variegata, Leucaena leucocephala, Mimosa pudica, Peltophorum pterocarpum, Pithecellobium dulce, Poinciana pulcherrima, Pterocarpus macrocarpus, Samanea saman, Sesbania grandiflora, Tamarindus indica*	Roots, rhizosphere	Mingma et al., 2014	Biocontrol
*Microbacterium*	*Trichilia elegans*	Leaves	Rhoden et al., 2015	Not determined

*The data presented is based on the references provided in column 4*.

* *Frankia is known to induce root nodules on a diverse group of angiosperm plants termed actinorhizals*.

In recent years, metagenomic analyses have been used to determine the bacterial communities of several agriculturally important crops. These studies have shown that actinobacteria are present in many of these plant microbiomes. Okubo et al. (2014) demonstrated that while the shoots of two field-grown rice cultivars collected in Nipponbare and Kasalath were dominated by *Alphaproteobacteria* (approximately 52%), the actinobacterial populations made up to 15% of the bacterial community structure. The characterization of the natural microbiome of *Vitis vinifera* leaves in Portugal reported a high diversity of proteobacteria, firmicutes, and actinobacteria, where the latter group accounted for approximately 19% of the microbial community composition and members of the families *Corynebacteriaceae, Microbacteriaceae*, and *Kineosporiaceae* were identified (Pinto et al., 2014).

A recent study to determine the bacterial communities of *Olea europaea* L. cultivars collected from different regions in the Mediterranean basin also confirmed the presence of actinobacterial populations on the olive leaf endosphere. An interesting conclusion of this work was that soil, climate conditions, and geographical distances had little effect on the endophytic microbial community composition (Müller et al., 2015). In another study, the root microbiota of *Lactuca sativa* cultivars and its wild ancestor *Lactuca serriola* were analyzed, the lettuce microbiota was dominated by *Proteobacteria* and *Bacteriodetes*, but *Chloroflexi* and *Actinobacteria* were also abundant (Cardinale et al., 2015). The composition of the actinobacterial population included members of the families *Micromonosporaceae* and *Nocardioaceae* but also the genera *Actinoplanes, Aeromicrobium, Arthrobacter, Demequina*, and *Streptomyces*. Interestingly, the domesticated cultivar (*L. sativa*) was richer in species diversity than its wild counterpart *L. serriola*. Unfortunately for most of the above studies, the function of these microorganisms on their host plants is unknown. In the case of lettuce, which is one of the raw foods widely consumed, it has been suggested that bacteria present in the plant's root such as *Streptomyces*, may serve as biological control agents by producing antibiotics to eliminate potential human pathogens (e.g., enterobacteria) (Cardinale et al., 2015).

Several soil microbiomes related to *Andropogon gerardii, Schizachyrium scoparium, Lespedeza capitata*, and *Lupinus perennis* grown in communities which varied in plant richness (1–16 species) were determined (Bakker et al., 2014). In this study the antagonistic activity and community structure of *Streptomyces* populations was assessed in relation to the species plant richness. The authors reported that the diversity and richness of bacterial and *Streptomyces* communities displayed different relationships with biotic and abiotic soil characteristics, therefore influencing bacterial communities.

The roots, leaves, and stems are the main plant tissues that have been screened for the presence of bacteria, however, nitrogen fixing nodules produced by legumes and actinorhizal plants are also an important reservoir of microorganisms. Nodules are rich in nutrients and therefore can also be colonized by bacteria unrelated to rhizobial or *Frankia* symbiotic nitrogen fixation.

Actinobacterial strains identified in the genera *Agromyces, Curtobacterium, Microbacterium, Micromonospora*, and *Streptomyces* have been reported from nodule tissues (Sturz et al., 1997; Trujillo et al., 2006, 2007, 2010; Zakhia et al., 2006; Muresu et al., 2008; Stajkoviæ et al., 2009; Deng et al., 2011; Hoque et al., 2011; Li et al., 2011; Carro et al., 2012a). Of these, the genera *Microbacterium* and *Micromonospora* were the most frequently isolated. Host plants inoculated with some of these strains showed better growth and development in comparison with non-inoculated controls suggesting a beneficial effect (Trujillo et al., 2010, 2014b; Deng et al., 2011; Martínez-Hidalgo et al., 2014). However, our knowledge about these new plant-microbe interactions is still very poor given the limited data currently available.

In light of their ecological importance, *Frankia* as a provider of nitrogen to actinorhizal plants, and *Streptomyces* as a plant pathogen for important crops such as potato, these bacteria have been under research for many decades, but this is not the case for most of other reported plant-actinobacteria interactions. However, in the last 10 years the interaction *Micromonospora*-nitrogen fixing plants is gaining attention due its potential application in downstream biotechnological applications, especially in the area of agriculture. In the following sections we will provide a general overview on the past and present status of *Micromonospora* and its close interaction with legumes and actinorhizal plants.

## *Micromonospora* and nitrogen fixing nodules: A universal plant-microbe interaction?

**Figure 1:**
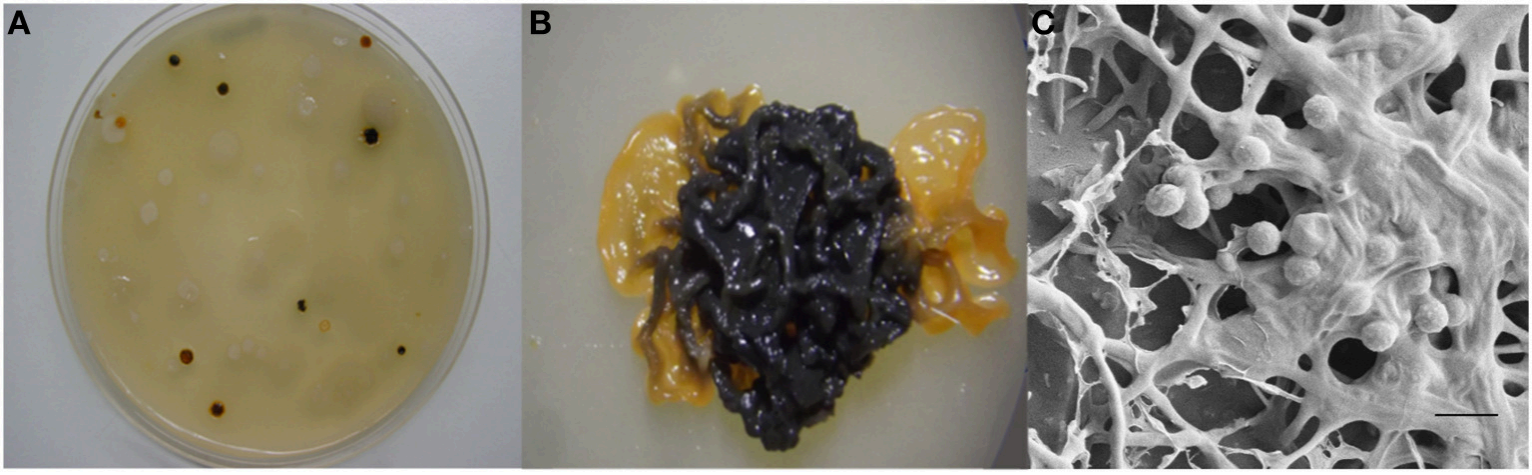
**Morphological features of *Micromonospora***. **(A)** *Micromonosporae* isolates recovered from a nitrogen fixing nodule. **(B)** 14 day old colony producing brown-black spores. **(C)** Scanning electron micrograph of a mucous mass of spores. Bar, 1 μm (Carro, 2009; Alonso de la Vega, 2010).

The actinobacterium *Micromonospora* was first described in 1923 (Ørskov, 1923). The first strains originated from soil and Jensen (1932) pointed out the importance of this microorganism in this niche. This bacterium belongs to the family *Micromonosporaceae* and includes aerobic, filamentous, spore-producing and mesophilic microorganisms. *Micromonospora* colonies are usually pigmented and range in color from orange, red, or brown. In many old cultures a brown-black, or black mucous mass of spores is observed. The formation of single spores is the main morphological characteristic of the genus *Micromonospora*; however, spores are also produced in dense clusters on the surface or completely embedded in the substrate mycelium (Figure 1) (Genilloud, 2012; Trujillo et al., 2014a).

The presence of *Micromonospora* has been reported from many geographical sites worldwide and although soil is the most frequent source of isolation, marine, aquatic sediments and mangrove environments are also inhabited by this microorganism (Maldonado et al., 2009; Genilloud, 2012; Trujillo et al., 2014a). In recent years *Micromonosporae* have been reported as major components of nitrogen fixing root nodules of both leguminous and actinorhizal plants (Valdés et al., 2005; Trujillo et al., 2006, 2007, 2010; Garcia et al., 2010; Carro et al., 2012a, 2013a). Isolation of *Micromonospora* strains from internal nodular tissues has been reported from the legumes *Arachis hypogaea, Cicer arietinum, Glycine max, Lens culinaris, Lupinus angustifolius, Lupinus gredensis, Medicago sativa, Melilotus* sp., *Mucuna* sp., *Ononis* sp., *Ornithopus* sp., *Phaseolus* sp.*, Trifolium* sp., and *Vicia* sp. The isolation of *Micromonospora* strains usually requires selective isolation procedures to favor its slow growth, however, in all the above examples, the same isolation protocol as that used for the isolation of rhizobia was applied (Cerda, 2008; Rodríguez, 2008; Carro, 2009; Alonso de la Vega, 2010; Trujillo et al., 2010).

Actinorhizal plants that have been sampled to date in Mexico, Spain, Canada, and France include the species *Alnus viridis, Casuarina equisetifolia, Coriaria myrtifolia, Elaeagnus x ebbingei, Hippophae rhamnoides, Myrica gale*, and *Morella pensylvanica* (Valdés et al., 2005; Trujillo et al., 2006; Carro et al., 2013a). Except for the study of Valdés et al. (2005), the isolation of *Micromonospora* from actinorhizal nodules also followed the same isolation protocols as that of legumes, using yeast-mannitol agar as isolation medium (Vincent, 1970). Currently our group maintains a collection of ~2000 isolates recovered from diverse legume and actinorhizal plants species collected in Spain, France, Germany, Ecuador, Nicaragua, and Australia but our hypothesis is that *Micromonospora* is also present in those plant species which have not been sampled to date. In the case of legumes, the above examples indicate how *Micromonospora* had been largely overlooked in this niche due to its slow growth as compared to rhizobial strains which can be readily recovered from isolation plates after 3–5 days while *Micromonospora* strains usually appear after 7–10 days on the same plates. While the work carried by Carro et al. (2013a) strongly suggests that this microorganism is also a normal occupant of actinorhizal nodules. Thus, the systematic recovery of *Micromonospora* populations strongly suggests that this bacterium closely interacts with the host plant and nitrogen-fixing bacteria occupying the same niche.

The biogeographical and species distribution of *Micromonosporae* isolated from nitrogen fixing nodules of legumes and actinorhizal plants sampled hitherto is presented in Table 2.

**Table 2:** **Biogeographical and species distribution of *Micromonosporae* in nitrogen fixing nodules of legumes and actinorhizal plants sampled**.

**Host plant (Legumes)**	**Common name**	**Geographical origin**	**Closest species identification (16S rRNA gene)**	**References**
*Arachys* sp.	Peanut	Nicaragua	*M. chaiyapumensis, M. endolithica*	Cerda, 2008
*Cicer arietinum*	Chickpea	Spain	ND	Trujillo et al., 2010
*Glycine max*	Soy	Nicaragua	ND	Trujillo et al., 2010
*Lens culinarium*	Lentil	Spain	ND	Trujillo et al., 2010
*Lupinus angustifolius*	Blue lupine	Spain	*M. aurantiaca, M. auratinigra, M. chaiyapumensis, M. coriariae, M. coxensis, M. echinospora, M. fulviviridis, M. lupini, M. matsumotoense, M. narathiwatensis, M. olivasterospora, M. sagamiensis, M. saelicesensis*	Trujillo et al., 2007; Rodríguez, 2008; Alonso de la Vega, 2010
*Lupinus gredensis*	Lupine	Spain	*M. chaiyapumensis, M. chersina, M. coxensis, M. echinofusca, M. echinospora, M. lupini, M. olivasterospora, M. saelicesensis, M. viridifaciens*	Alonso de la Vega, 2010
*Lupinus* sp.	Lupine	Germany	*M. saelicesensis*	Trujillo et al., 2010
*Medicago* sp.	Alfalfa	Australia, Spain	*M. aurantiaca, M. chokoriensis, M. lupini, M. saelicesensis, M. schwarzwaldensis, M. tulbaghiae, M. viridifaciens*	Martínez-Hidalgo et al., 2014
*Mucuna* sp.	Mucuna	Ecuador	ND	Trujillo et al., 2010
*Ononis* sp.	–	Spain	ND	Trujillo et al., 2010
*Ornithopus* sp.	–	Spain	ND	Trujillo et al., 2010
*Phaseolus vulgaris*	Bean	Nicaragua	*M. chaiyapumensis, M. chersina, M. endolithica*	Cerda, 2008
*Pisum sativum*	Sweet pea	Spain	*M. aurantica, M. auratinigra, M. chaiyapumensis, M. chersina, M. coerulea, M. coriariae, M. coxensis, M. fulviviridis, M. lupini, M. matsumotoense, M. pattaloongensis, M. saelicesensis, M. sagamiensis„ M. siamensis*	Carro, 2009; Carro et al., 2012a
*Trifolium* sp.	Clover	Spain	ND	Trujillo et al., 2010
*Vicia* sp.	Vetch	Spain	ND	Trujillo et al., 2010
**HOST PLANT (ACTINORHIZALS)**				
*Alnus glutinosa*	Alder	France	*M. cremea, M. coxensis, M. lupini, M. matsumotoense, M. olivasterospora, M. saelicesensis, M. siamensis*	Carro et al., 2013a
*Alnus viridis*	Alder	France	*M. chokoriensis, M. coriariae, M. lupini, M. matsumotoense, M. pisi, M. rifamycinica, M. saelicesensis*	Carro et al., 2013a
*Casuarina equisetifolia*	Coast sheoak	Mexico	*M. aurantiaca*	Valdés et al., 2005
*Coriaria myrtifolia*	Redoul	Spain, France	*M. coriarie, M. saelicesensis, M. peucetia*	Trujillo et al., 2006; Carro et al., 2013a
*Elaeagnus x ebbingei*	–	France	*M. aurantiaca, M. auratinigra, M. chaiyaphumensis, M. coriariae, M. coerulea, M. cremea, M. coxensis, M. equina, M. lupini, M. matsumotoense, M. mirobrigensis, M. peucetia, M. saelicesensis, M. siamensis*	Carro et al., 2013a
*Hippophae rhamnoides*	Sandthorne	France	*M. chaiyapumensis, M. chersina, M. coxensis, M. equina, M. lupini, M narathiwatensis, M. saelicesensis, M. siamensis, M. viridifaciens*	Carro et al., 2013a
*Morella pensylvanica*	–	France	*M. coriariae, M. cremea, M. olivasteraspora, M. peucetia, M. saelicesensis*	Carro et al., 2013a
*Myrica gale*		Canada	*M. lupini, M. tulbaghiae*	Carro et al., 2013a

## Distribution, localization and genetic diversity of *Micromonospora* in nitrogen fixing nodules

The distribution of *Micromonospora* strains in the nitrogen fixing nodules sampled so far indicate that its distribution is not homogeneous and it varies from nodule to nodule and plant to plant (Trujillo et al., 2010; Carro et al., 2012a).

The distribution pattern of *Micromonospora* in *Lupinus* spp. is highly variable with no isolates for some nodules to as many as approximately 30 (Alonso de la Vega, 2010; Trujillo et al., 2010). Variation is also reported from plant to plant and from different nodules of the same plant (Trujillo et al., 2010). A comparison of the species *Lupinus angustifolius* and *Lupinus gredensis* collected in the same geographical area in Spain, indicated that 67 and 60% of the plant samples screened (17 in total) contained the target microorganism, respectively. Out of the 45 nodules chosen for isolation, 95 *Micromonospora* strains were recovered, 74 from *L. angustifolius* and 21 from *L. gredensis*. Interestingly, 48% of the nodules did not appear to contain any *Micromonospora* strains (Alonso de la Vega, 2010).

In terms of the bacterial species distribution, *Micromonospora saelicesensis* and *Micromonospora lupini* were the most abundant, nevertheless the diversity determined on the basis of 16S rRNA gene sequencing was very high (Alonso de la Vega, 2010; Trujillo et al., 2010). These authors also screened lupine plants at different growth stages which corresponded to young, maximum growth, and flowering plants. In this case, the number of bacteria increased in parallel to the plant growth and decreased as the plants became old.

As for the legume *Pisum sativum*, a similar pattern of distribution was observed. However, for this plant, at least one *Micromonospora* strain was recovered from every nodule sampled (Carro et al., 2012a). It is also important to note that while lupine plants were collected in the field, all *Pisum sativum* samples originated from cultivation fields where chemical fertilizers are applied periodically (Carro et al., 2012a).

In a recent study, Carro et al. (2013a) screened several actinorhizal plants and recorded the number of *Micromonospora* strains and species found. *Micromonospora* strains were recovered from all plants sampled, and, as in the case of legumes, the number of isolates also varied significantly. High numbers of *Micromonospora* strains were isolated from *Alnus, Elaeagnus*, and *Hippophae* nodules, while the number of isolates was much lower in *Myrica, Morella*, and *Coriaria* nodules. Similarly to legumes, most isolates were related to *M. saelicesensis* and *M. lupini* but *M. coriariae* was also isolated in high numbers. The latter species was first reported from *Coriaria myrtifolia* nodules (Trujillo et al., 2006).

The first *Micromonospora* strains isolated from nitrogen fixing nodules were considered contaminants because it was assumed that the spores produced by this microorganism were soil contaminants that had resisted the sterilization protocols. However, the absence of other fast-growing sporulating microorganisms, e.g., fungi or *Streptomyces* strongly indicated that the strains had originated from the internal plant tissues (Trujillo et al., 2010). Applying fluorescent *in situ* hybridization (FISH) and transmission electronic microscopy (TEM), *Micromonospora lupini* Lupac 08 was localized inside the nodular tissues of lupin suggesting a close interaction between the host plant and the bacterium (Rodríguez, 2008; Trujillo et al., 2010). Further experiments using a *Micromonospora* strain tagged with green fluorescent protein to trace the microorganism *in planta* are in the process of completion.

**Figure 2:**
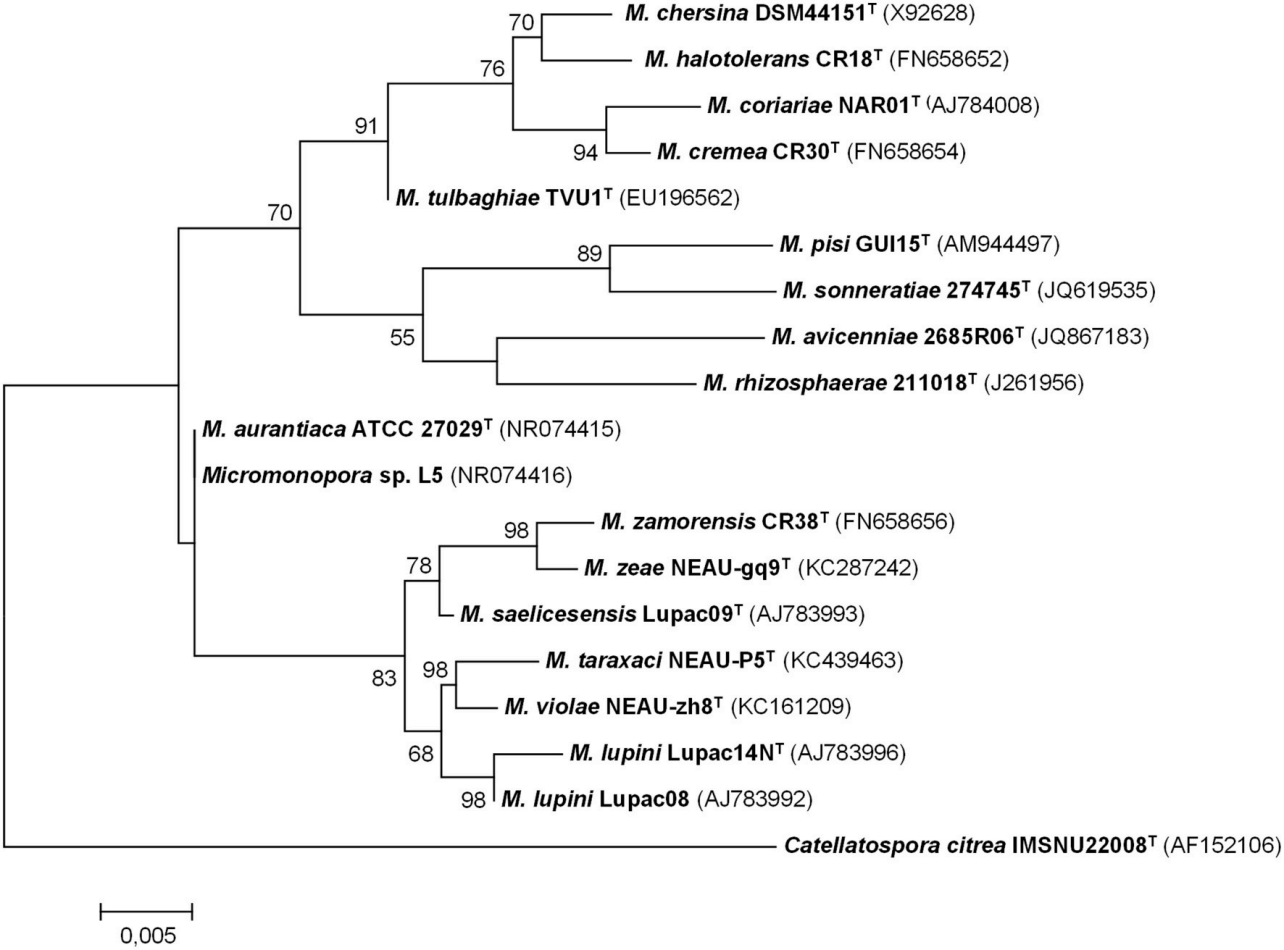
**Maximum-likelihood phylogenetic tree based on 16S rRNA gene sequences of *Micromonospora* species isolated from plant material and rhizospheric soil**. There were 1408 nucleotides in the final dataset. Analyses were carried in MEGA 6 software. Bar indicates 0.005 substitutions per nucleotide position (Based on references provided in Table 2).

The degree of genetic variation of *Micromonospora* strains recovered from the nitrogen-fixing nodules of various plants was analyzed using several molecular typing techniques (e.g., BOX–PCR, ARDRA, RFLP, RAPDS) (Cerda, 2008; Carro, 2009; Alonso de la Vega, 2010; Trujillo et al., 2010; Carro et al., 2012a; Martínez-Hidalgo et al., 2014). Highly diverse genetic fingerprint profiles were found among the isolates studied, indicating that they were not clones; the diversity found was unexpectedly high considering that in some cases, the strains analyzed were isolated from the same nodule (Alonso de la Vega, 2010). Subsequently, taxonomic studies carried for some of these isolates confirmed that many of these bacterial strains represented new species and include *Micromonospora coriariae* (Trujillo et al., 2006); *Micromonospora lupini* and *Micromonospora saelicesensis* (Trujillo et al., 2007); *Micromonospora pisi* (Garcia et al., 2010); *Micromonospora cremea, Micromonospora zamorensis*, and *Micromonospora halotolerans* (Figure 2). The latter three strains were isolated from the rhizospheric soil of the sampled plants (Carro et al., 2012b, 2013b).

The species *M. saelicesensis* is the most frequently isolated from the nodule tissues in both legume and actinorhizal plants, followed by the species *M. lupini* (Cerda, 2008; Carro, 2009; Alonso de la Vega, 2010; Trujillo et al., 2010; Carro et al., 2012a). Furthermore, the number of new species found in this niche also appears to be very high as commented above. To expand the taxonomic studies of the genus *Micromonospora*, Carro et al. (2012a) carried out a multilocus sequence analysis study based on five loci and over 90 *Micromonospora* isolates recovered from the rhizosphere and plant tissues (nodules) of *P. sativum*. These studies were complemented with DNA-DNA hybridization analyses to confirm the high diversity at the species level (Carro et al., 2012a) and revealed that many of the new isolates represent new species (Carro et al., 2012b, 2013b).

## Genome features of *Micromonospora* isolated from nodules

Very few *Micromonospora* strains have been sequenced. At present, only five *Micromonospora* genomes are available in the public databases: *Micromonospora* sp. strain L5 and *M. lupini* Lupac 08 and isolated from nodules of *Casuarina equisetifolia* and *Lupinus angustifolius*, respectively (Alonso-Vega et al., 2012; Hirsch et al., 2013). The remaining are the soil isolates *Micromonospora aurantiaca* ATCC 27029^T^ (Hirsch et al., 2013), *Micromonospora* sp. ATCC 39149 (Accession No. GCF_000158815.1) and *Micromonospora carbonacea* JXNU-1 (Jiang et al., 2015). Several genomic characteristics of the strains are presented in Table 3. Actinobacterial genomes are usually larger than those of most other bacteria, e.g., proteobacteria and *Micromonospora* is no exception, the currently available genomes range from 6.9 to 7.3 Mb and share a similar GC content (72–74%).

**Table 3:** **Genomic features of sequenced *Micromonospora* strains available in the databases**.

**Feature**	***M. lupini***	***M. aurantiaca***	***Micromonospora***	***Micromonospora***	***Micromonospora***
	**Lupac 08**	**ATCC 27029^T^**	**sp. L5**	**sp. ATCC 39149**	***carbonacea* JXNU-1**
Size (Mb)	7.3	7	6.9	6.8	7.6
GC%	72	73	73	72	74
rRNA Operon	10	9	9	6	7
tRNA	77	52	53	51	50
CDS number	7054	6676	6617	5633	6247
Genes in COGs (%)	70.20%	68.30%	69%	nd	nd

*nd, not determined*.

The genome sequence of strain Lupac 08 was determined to identify genomic traits potentially involved in this plant-microbe interaction (Alonso-Vega et al., 2012; Trujillo et al., 2014b). The annotated genome disclosed various traits potentially involved in the capacity of this bacterium to alternate a lifestyle as a saprophyte in the soil and as an endophyte inside the root nodules (Trujillo et al., 2014b). The genome of strain Lupac 08 has a circular chromosome of 7.3 Mb with a GC content of 71.9% and lacking plasmids. A total of 10 rRNA genes were identified, specifically 3 5S rRNA, 4 16S rRNA, and 3 23S rRNA genes. In addition 77 tRNA genes were predicted (Alonso-Vega et al., 2012). Approximately, 62% (4338 CDSs) of the genes were assigned a biological function while 38% were annotated hypothetical open reading frames with unknown biological activities (Alonso-Vega et al., 2012). The genome of *Micromonospora* sp. L5 is smaller, 6.9 Mb, a GC content of 72.9% and 6332 open reading frames (Hirsch et al., 2013). This strain is highly related to *M. aurantiaca* ATCC 27029^T^ and average nucleotide identity values (ANI) of their genomes strongly suggest that *Micromonospora* sp. L5 belongs to this species. The number of tRNAs identified in *Micromonospora* sp. L5 is 52 (Hirsch et al., 2013) which is much lower when compared to the 77 tRNAs identified in *M. lupini* 08. Indeed, the latter strain has one of the largest numbers of tRNAs reported for actinobacteria sequenced to date. The number of rRNA and tRNA genes in a genome appear to be correlated and is an indication of positive selection related to the time of response of a bacterium to adapt to its environment (Dethlefsen and Schmidt, 2007; Yano et al., 2013).

The core genome of the strains *M. lupini* Lupac 08, *M. aurantiaca* ATCC 27029^T^ and *Micromonospora* sp. L5 was determined and the results indicated that the strains shared a common gene pool of only approximately 32% suggesting a high degree of genomic diversity (Trujillo et al., 2014b). As expected, the strains *M. aurantiaca* and *Micromonospora* L5 with 85% genome similarity confirm their close relationship. *M. lupini* on the other hand appears to be very different, with 66.6% of its genome being strain specific. As more *Micromonospora* genomes are sequenced the core genome should be better defined.

A number of genomic traits that probably participate in the plant/soil life style of endophytic *Micromonospora* include transport and secretion systems. Several genes coding for transport and secretion systems which may be involved in plant colonization were also identified. The number of transporters is slightly higher in *M. lupini* Lupac 08 than in *Micromonospora* L5, and included ATP dependent (mainly of the ABC family type), ion channels, PTS (phosphotransferase) and secondary transporters (Trujillo et al., 2014b).

## *Micromonospora* lupini Lupac 08: A friendly bacterium highly equipped with plant cell wall degrading enzymes

*Micromonosporae* are well-known for their capacity to produce high numbers of cellulases, these enzymes very likely contribute to the turn-over of decayed material in different habitats (de Menezes et al., 2008, 2012). However, the presence of high numbers of these molecules and other plant-cell wall degrading enzymes in beneficial endophytic bacteria is usually very low (Krause et al., 2007; Mastronunzio et al., 2008; Taghavi et al., 2010; Pujic et al., 2012).

**Figure 3:**
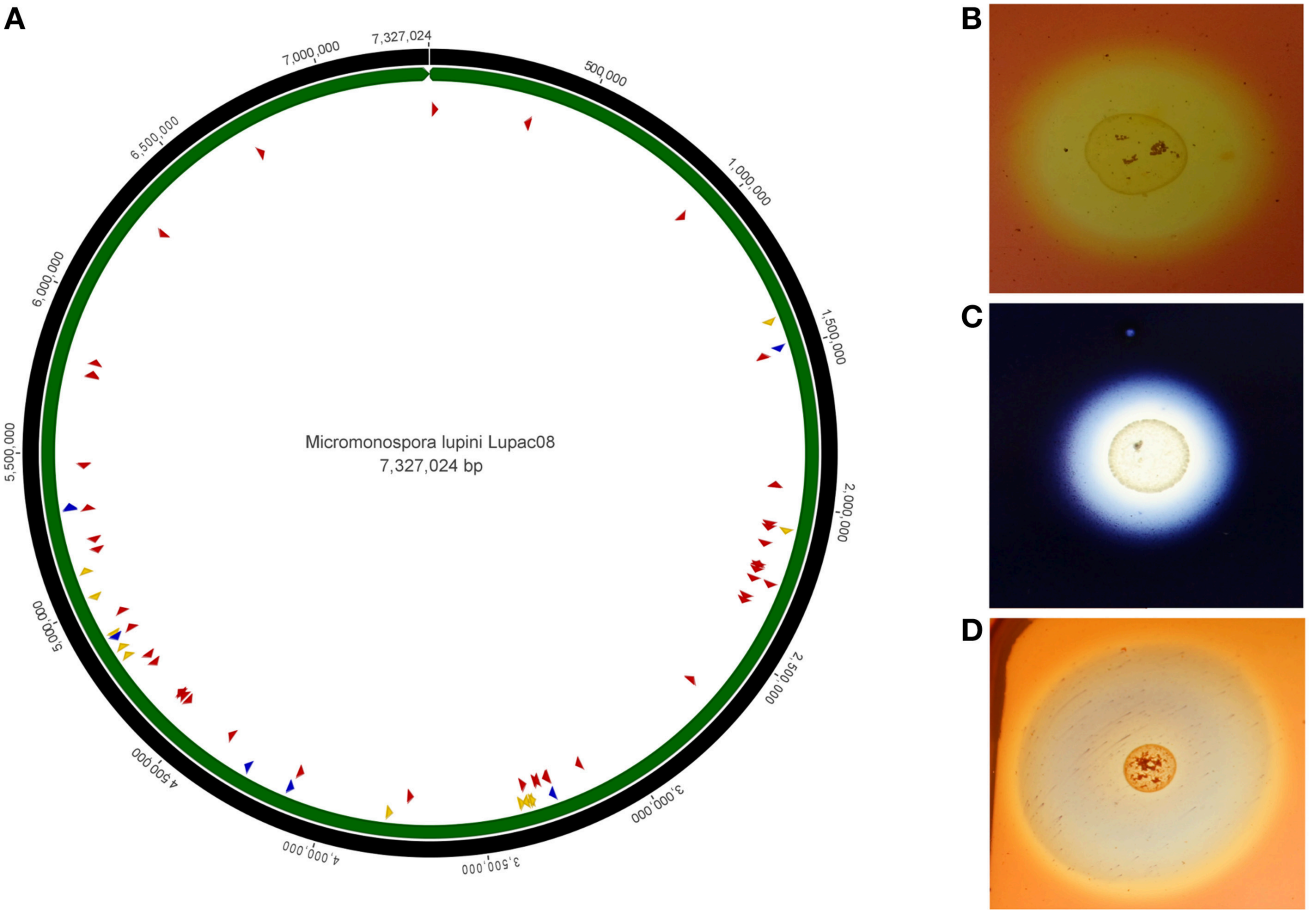
**Circular genome representation of *Micromonospora lupini*, Lupac 08. (A)** Distribution of various plant-cell wall hydrolytic enzyme loci. Red, cellulases, and cellulose-binding sites; blue, pectinases; yellow, xylanases. **(B)** *In vitro* cellulase degradation. **(C)** *In vitro* starch degradation. **(D)** *In vitro* xylanase degradation (Based on Trujillo et al., 2014b).

The genome of strain Lupac 08 contains a high number of genes encoding enzymes potentially involved in plant cell wall degradation. Approximately 10% of the genome codes for carbohydrate metabolism, and almost 200 out of the 685 genes have a putative hydrolytic function. Hydrolytic activities for cellulose, pectin, starch, and xylan, were confirmed in the laboratory and indicate that this strain could degrade plant cell wall components in a way similar to that of phytopathogen bacteria (Trujillo et al., 2014b). Plant-polymer degrading enzymes are thought to be involved in internal plant colonization (Compant et al., 2005). Plant pathogenic fungi and bacteria usually enter plant tissues by degrading plant cell wall components using several hydrolases which include cellulases and endoglucanases. On the other hand, genome data show that non-pathogenic (endophytic or symbiotic) microorganisms contain a low set of plant-polymer degrading enzymes (Krause et al., 2007; Mastronunzio et al., 2008; Taghavi et al., 2010). In the case of *M lupini*, the genome of this microorganism revealed a high number of hydrolytic enzymes (e.g., cellulases, xylanases, endoglucanases) with the potential to degrade plant tissues (Figure 3). However, green-house experiments show that when host plants are inoculated with strain Lupac 08 no damage is produced. On the contrary, *M. lupini* stimulates nodulation and plant growth (Cerda, 2008; Trujillo et al., 2014b). Therefore, if the plant does appear to be negatively affected by these enzymes, what is their potential function when the bacterium interacts with its host plant? Our group is currently working on this subject, some of the loci, especially those related to cellulose metabolism may participate in other processes such as cellulose biosynthesis (Robledo et al., 2008, 2012; Mba Medie et al., 2012). Several genes coding for plant cell-wall degrading enzymes were also located in the genome of *Micromonospora* sp. L5 (Hirsch et al., 2013). Similarly to strain Lupac 08, target substrates include cellulose, hemicellulose, pectin, starch, and xylan, however, the number of loci involved in carbohydrate transport and metabolism are slightly lower in strain L5 (8.9%), as compared to strain Lupac 08 (9.7%) (Trujillo et al., 2014b).

Bacterial endophytic colonization is still a poorly understood process, in part because it is very complex. For microorganisms that colonize the roots, plant exudates appear to play a crucial role (Badri et al., 2009). Molecules present in root exudates may serve as carbon sources for microorganisms and therefore, these are attracted to the plant roots (Shidore et al., 2012). Thus, plant exudates may act as signals that influence the ability of a bacterium to colonize the root or survive in the rhizosphere. These signals may induce the alteration of specific gene expression patterns in the bacterium, which in turn may influence its interaction with the plant (Morrissey et al., 2004; Mark et al., 2005; Shidore et al., 2012). While it is considered that plant exudates affect the behavior of rhizospheric microorganisms, our knowledge as to how these molecules influence bacterial gene expression is still very limited (Mark et al., 2005). Furthermore, it is not known how these altered bacterial genes affect the plant-microbe interaction process and only a few studies are available (Morrissey et al., 2004; Mark et al., 2005; Shidore et al., 2012).

In the case of the *Micromonospora*-plant interaction, it could be that the plant's root exudates might be involved in the repression of hydrolytic enzyme genes (e.g., cellulases, xylanases, etc.) from the bacterium which, if expressed during its interaction with the plant would be detrimental upon infection. The effect on *Azoarcus* sp. gene expression upon exposure to plant root exudates was recently reported (Shidore et al., 2012). This study concluded that the genes expressed by *Azoarcus* strain BH72 upon exposure to the plant's root exudates influenced the colonization of the roots (Shidore et al., 2012). In this sense, the genome of *M. lupini* contains many regulatory genes located near plant cell wall degrading loci suggesting that these genes are under strong regulation, which in turn, may be directly related to the surrounding environment, soil, or plant tissues (Trujillo et al., 2014b).

## *Micromonospora*, a plant growth promoter with wide application in agriculture

Plant growth promoting bacteria (PGPB) are defined as soil bacteria that facilitate plant growth and are often found in association with plant roots, leaves, flowers, or within plant tissues. Many of these bacteria are found in the plant rhizoplane and rhizosphere but other are endophytic and able to colonize the internal plant tissues (Glick, 2015). Plant growth promoting bacteria have been reported to positively affect plants in a number of ways, directly by facilitating resource acquisition (e.g., nitrogen fixation, phosphorous, iron) or controlling plant hormone levels, or indirectly by lowering the inhibitory effects of plant pathogen microorganisms (e.g., biocontrol agents).

The current data about the interaction of *Micromonospora* with legume and actinorhizal plants is limited, and therefore the bacterium's ecological role inside the roots nodules and its interaction with the nitrogen fixing bacteria (rhizobia/*Frankia*) is unknown. Plant co-inoculation studies indicate that *Micromonospora* acts as a plant growth promoting bacterium with a positive effect on the plant (Martínez-Hidalgo et al., 2014; Trujillo et al., 2014b). Nodulation and nitrogen tests were carried out on *Lupinus* and *Phaseolus*, these studies showed that *Micromonospora* is not able to induce nodules or fix nitrogen but a positive effect on the growth of the plant was observed by an increase in the number of nodules and the height of the plants which had been inoculated with both microorganisms when compared to the plants treated with only one of the two strains (Cerda, 2008). Furthermore, when *Micromonospora* and the nitrogen-fixing bacterium (*Bradyrhizobium* or *Rhizobium*, respectively) were grown together, they were compatible and did not inhibit the growth of each other. Interestingly, *Micromonospora* did inhibit the growth of several *Frankia* strains; furthermore the latter strains came from different plant species (Carro et al., 2013a). On the other hand no inhibition was observed between *Micromonospora* and *Frankia* when the strains originated from the same plant (Carro et al., 2013a).

Studies carried out with *Trifolium* plants yielded similar results. *Micromonospora lupini* Lupac 08 stimulated plant growth when it was co-inoculated with *Rhizobium* sp. on clover plantlets and these were grown in a greenhouse (Trujillo et al., 2014b). In general, the number of nitrogen-fixing nodules increased in plants treated with both bacteria as compared to the plants inoculated only with the *Rhizobium* strain. Overall, the plants inoculated with both bacteria exhibited better growth and increased shoot length compared to single-strain treatments (Trujillo et al., 2014b).

Solans (2007) studied the plant promotion effect of three actinobacterial strains isolated from the plant species *Discaria trinervis* which included a *Micromonospora* strain. The inoculation experiments of *D. trinervis* grown in glass tubes with vermiculite-sand was done using pure mycelia suspensions and/or supernatants obtained from the actinobacterial cultures grown for 8 days. Plants inoculated with mycelium plus supernatant from *Micromonospora* strain BCRU-MM18 had a higher shoot length than the control plants and it was proposed that this effect was probably due to the presence of several plant hormones such as zeatin, IAA, and gibberellic acid. Further studies confirmed that strain BCRU-MM18 produced significant amounts of IAA (9.03 ng/ml), giberellic acid (9.03 ng/ml), and zeatin (270 μg/ml); in all cases these amounts were higher than those produced by the nitrogen fixer *Frankia* sp. BCU110501 (Solans et al., 2011). The same *Micromonospora* strain (BCRU-MM18) was co-inoculated in *Medicago sativa* which had also been inoculated with the nitrogen fixer *Sinorhizobium meliloti* in the presence of high nitrogen content. Unexpectedly, a promotion of nodulation was observed despite the high amounts of nitrogen present (7 mM) which usually inhibit nodulation (Solans et al., 2009). The above studies showed the positive effect that *Micromonospora* had on the symbiosis of both leguminous and actinorhizal plants, especially in increasing nodulation rates.

Recently, *Micromonospora* strains isolated from wild alfalfa plants collected in several sites in Spain were studied for their plant growth and nutrient content effect on this legume. Selected strains significantly increased the nodulation of *Medicago* sp. inoculated with *Ensifer meliloti* and also the plant's efficiency for nitrogen uptake. Furthermore, aerial growth, shoot-to-root ratio and increase in levels of key nutrients was also reported (Martínez-Hidalgo et al., 2014). These authors also discussed the importance of choosing the most effective strains.

The wide distribution of *Micromonospora* among nitrogen fixing plants (both legumes and actinorhizals) differs from that of rhizobia or *Frankia* which are limited to a narrow host range of legumes and angiosperms, respectively. The capacity of infection by *Micromonospora* with a positive effect for its host plant may be regarded as an advantage for downstream biotechnological applications and the potential to use this bacterium as a plant growth promoter in combination with rhizobia or *Frankia*.

## The *micromonospora* metabolome and its potential role in plant-microbe communication signals

Microbial secondary metabolites have been the subject of many research projects, mainly with the aim to discover new compounds with biotechnological application (Miao and Davies, 2010; Genilloud, 2014). However, our knowledge about the ecological role of these compounds is very limited. It is proposed, that in the environment, these natural products serve as allelochemicals and signaling molecules to communicate with organisms, in this case, with the plant (Badri et al., 2009). Udwary et al. (2011) recently reported the identification of several biosynthetic gene clusters coding for secondary metabolites in the genome of *Frankia*. In this work, it was proposed that some of these compounds could function as communication molecules to establish the symbiotic interaction between *Frankia* and the host plant (Udwary et al., 2011). The potential role of lectins produced by *Frankia alni* ACN14a to permit binding of the bacterial cells to the roots of the host plant was suggested by Pujic et al. (2012). In another study, a hybrid (PKS)/NRPS protein produced by *Trichoderma virens* was proposed to induce the defense mechanisms of maize (Mukherjee et al., 2012).

Moreover, Conn et al. (2008) demonstrated that culture filtrates obtained from *Micromonospora* sp. strain EN43 isolated from healthy wheat tissues were able to induce several plant defense systems in *Arabidopsis thaliana*. When the bacterium was grown in a minimal medium, the culture filtrate applied to the plant induced the systemic acquired system pathway; however, when grown in a complex medium, the jasmonic acid/ethylene pathway was activated (Conn et al., 2008). Based on these results, the authors suggested that different metabolites were produced under the two conditions tested and that these compounds were responsible for the activation of the different defense mechanisms in the plant (Conn et al., 2008). In addition, it was also proposed that a physical contact of the bacterium and the plant may be required for the defense mechanisms to be activated. Overall, the above examples show the potential ecological role of secondary metabolites in plant-microbe interactions.

The information derived from sequenced actinobacterial genomes have revealed that these microorganisms have the biosynthetic potential to make far more natural products than was realized before genome sequences were available (Genilloud, 2014). Only a small fraction of endophytic bacteria have been characterized and they remain as an untapped resource of novel bioactive small molecules (Qin et al., 2011; Brader et al., 2014). As mentioned above, some of these metabolites are speculated to affect the physiological conditions of host plants including growth and disease resistance (Conn et al., 2008; Udwary et al., 2011). *Micromonosporae* strains are also a good source for obtaining natural products (Weinstein et al., 1963; Thawai et al., 2004; Antal et al., 2005; Anzai et al., 2010; Kyeremeth et al., 2014). In this sense, the model strain *Micromonospora lupini* Lupac 08 is no exception and a family of new anthraquinone molecules with antitumoral activity were isolated and identified (Igarashi et al., 2007, 2011). Moreover, 15 clusters involved in the biosynthesis of secondary metabolites were identified in the genome of *M. lupini* Lupac 08. These included siderophores, terpenes, butyrolactones, polyketides (PKS), non-ribosomal peptides (NRPS), chalcone synthases and bacteriocins. Approximately 7.4% of the genome was related to genes coding for secondary metabolites.

The production of siderophores by endophytic bacteria is suggested to promote plant growth by sequestering iron from the environment and providing the nutrient to the plant. Alternatively, plant growth promoting bacteria can protect plants by binding the available iron surrounding the roots and limiting access to the nutrient by phytopathogen microorganisms (Glick, 2015). Recently it was shown that a siderophore-producing endophytic streptomyces strain significantly increased root and shoot biomass as compared to a siderophore deficient mutant strain (Rungin et al., 2012). Furthermore, Misk and Franco (2011) reported the capacity of several endophytic siderophore producing *Streptomyces* strains to suppress root rot in chickpea produced by *Phytophtora*. In this case, the streptomycete strains were isolated from several legumes. Several gene loci related with the synthesis of siderophores were identified in the genome of *M. lupini* Lupac 08 and the strain was shown to produce these molecules in the laboratory (Trujillo et al., 2014b). Siderophores produced by *Micromonospora* may also contribute to the increased root and shoot biomass observed when host plants are inoculated with this bacterium (Martínez-Hidalgo et al., 2014; Trujillo et al., 2014b).

The characterization and identification of secondary metabolites produced by *Micromonospora* strains isolated from nitrogen fixing plants is at present reduced to three anthraquinones, lupinacidins A, B, and C (Igarashi et al., 2007, 2011). However, the genome of strain Lupac 08 revealed that other metabolites are potentially produced (e.g., terpenes, butyrolactones, polyketides, non-ribosomal peptides etc.). These compounds may act as communication molecules between the microorganism and the plant to allow bacterial colonization (Udwary et al., 2011). Alternatively, as suggested by other studies these metabolites may provide protection against pathogens, either by producing specific control agents or by activating plant defense systems (Conn et al., 2008). Furthermore, some metabolites may be necessary for nutrient uptake (Barry and Challis, 2009; Rungin et al., 2012) All these areas remain to be studied in the interaction *Micromonospora*-nitrogen fixing plants.

## Concluding remarks

Our knowledge of the interaction between *Micromonospora* with legumes and actinorhizal plants is in its infancy and a lot more work is required to fully understand this ecological process. Apart from the studies presented above, there is no other information regarding the molecular interaction between *Micromonospora* and its host plants and how it interacts with other bacteria present in the nitrogen fixing nodules. The current data is promising as it strongly suggests that *Micromonospora* provides a benefit to the plant. The genome of strain Lupac 08 revealed many features that make this microorganism an excellent candidate as a plant-growth promoter which could be applied to a large number of agriculturally important crops.

### Conflict of interest statement

The authors declare that the research was conducted in the absence of any commercial or financial relationships that could be construed as a potential conflict of interest.
